# The Effect of Low-Level Laser Therapy on Hearing

**DOI:** 10.1155/2013/916370

**Published:** 2013-04-23

**Authors:** Shawn S. Goodman, Ruth A. Bentler, Andrew Dittberner, Ian B. Mertes

**Affiliations:** ^1^Department of Communication Sciences & Disorders, The University of Iowa, Iowa City, IA 52242, USA; ^2^GN Resound North America, Chicago, IL 60646, USA

## Abstract

One purported use of low-level laser therapy (LLLT) is to promote healing in damaged cells. The effects of LLLT on hearing loss and tinnitus have received some study, but results have been equivocal. The purpose of this study was to determine if LLLT improved hearing, speech understanding, and/or cochlear function in adults with hearing loss. Using a randomized, double-blind, placebo-controlled design, subjects were assigned to a treatment, placebo, or control group. The treatment group was given LLLT, which consisted of shining low-level lasers onto the outer ear, head, and neck. Each laser treatment lasted approximately five minutes. Three treatments were applied within the course of one week. A battery of auditory tests was administered immediately before the first treatment and immediately after the third treatment. The battery consisted of pure-tone audiometry, the Connected Speech Test, and transient-evoked otoacoustic emissions. Data were analyzed by comparing pre- and posttest results. No statistically significant differences were found between groups for any of the auditory tests. Additionally, no clinically significant differences were found in any individual subjects. This trial is registered with ClinicalTrials.gov (NCT01820416).

## 1. Introduction

Low-level laser therapy (LLLT) has been practiced for over 20 years in Europe and has more recently been introduced in the United States as a treatment for pain and postsurgical tissue repair. It has been proposed that laser energy in the red and near-infrared light spectrum may aid in the repair of tissue damage. A proposed mechanism for this therapeutic effect is the stimulation of mitochondria in the cells to produce more energy through the production of adenosine triphosphate [1–3]. 

It has been postulated that LLLT may improve cochlear function. Animal studies have found that laser stimulation can induce anatomic and physiologic changes in the cochlea. Rhee et al. [4] reported that rat hair cells were repaired with LLLT following noise exposure. Wenzel et al. [5] found that laser stimulation increased basilar membrane stiffness (and therefore resonant frequency) in guinea pigs. The authors suggested that this could allow lower-frequency regions of the cochlea (where auditory function is typically less compromised) to respond to higher frequency sounds.

Studies in humans have investigated the effects of LLLT on both hearing loss and tinnitus, with equivocal results. Some studies have found an improvement in hearing thresholds and tinnitus symptoms (e.g., [6–10]), while others have found no significant effect of LLLT (e.g., [11–15]). The reason for the discrepancy in findings is not known, but likely involves multiple factors such as study design, subject characteristics, LLLT methodology, and outcome measures used to assess the effects of LLLT. 

Further research on the effect of LLLT on hearing in humans appears warranted. Although some studies showed improvements in hearing thresholds, no published study to date has examined the effect of LLLT on speech understanding and only one has examined the effect on cochlear function via otoacoustic emissions [11]. Pilot data from HearingMed (unpublished) found that LLLT improved word recognition scores in subjects with hearing loss relative to a placebo group, which motivated the current study. The purpose of the current study was to assess the effect of LLLT on hearing in terms of auditory sensitivity, speech understanding, and cochlear function. A randomized, double-blind, placebo-controlled trial design was implemented using the laser therapy protocol suggested by the HearingMed study.

## 2. Materials and Methods

### 2.1. Subjects

In order to accurately detect possible changes in hearing status due to laser treatment, it was necessary to avoid using subjects whose hearing might fluctuate due to other factors. Before potential subjects were enrolled in the study, they were asked a list of screening questions to determine eligibility. The questions were chosen to ensure stable hearing, as well as to address possible safety issues. All subjects were also required to have normal middle ear function, as assessed by 226 Hz tympanograms.

A total of 35 adult subjects were enrolled in the study. Two subjects withdrew from the study due to loss of interest and/or scheduling difficulty. The data from three additional subjects were not included in the analysis. One subject yielded unreliable audiometric and speech understanding data, speech scores could not be obtained from one subject with a profound hearing loss, and calibration problems compromised data from the third subject. Data from the remaining 30 subjects were included in the analyses. The experimental protocol was approved by the Institutional Review Board of The University of Iowa, and written informed consent was obtained from all participants.

### 2.2. Laser Device

An Erchonia EHL laser (Erchonia Medical, Inc.) was used to provide the laser stimulation. The device was a portable (9′′ × 5′′ × 1′′) unit that consisted of a hand-held probe and a main body. The probe contained two laser diodes. One diode produced light in the green part of the visible light spectrum (532 nm wavelength), and the other diode produced light in the red part of the visible light spectrum (635 nm wavelength). Both diodes produced energy levels of 7.5 mW (class IIIb). The laser beams from both diodes were dispersed through lenses to create parallel line-generated beams, rather than spots. The 532 nm light was constant, and the 635 nm light was pulsed, with frequencies of 15 and 33 Hz. The pulsing alternated between frequencies every 30 seconds. A second Erchonia EHL device served as the placebo. It was identical to the treatment device, except that the laser diodes were replaced with nonfunctioning standard light-emitting diodes. 

### 2.3. Study Design

#### 2.3.1. Groups

The study used three groups: treatment, placebo, and control. Subjects were pseudorandomly assigned to one of the three groups. Initial group assignment was random with occasional adjustment to ensure that the three groups were similar in terms of number of participants, female/male ratio, mean age of participants, and mean pure-tone audiometric thresholds. The composition of each group is shown in Table 1.

The treatment group received the laser treatment protocol (described in Section 2.4) using the functional laser device. The placebo group also received the laser treatment protocol, but using the nonfunctioning laser device. The control group made similarly timed visits to the laboratory but received no real or feigned “treatment.” The study used a repeated-measures design, with each subject taking a battery of pretests, followed by treatment (laser, sham laser, or nothing), followed by a battery of posttests. 

#### 2.3.2. Timeline

The pretest, treatment sessions, and posttest took place in three scheduled visits within a 7–10-day period for each subject. On the first visit, the pretests were administered, followed immediately by the treatment session. Subjects in the treatment group received laser treatment with the functioning laser device. Subjects in the placebo group received laser treatment with the nonfunctioning device. Subjects in the control group simply sat in a comfortable chair and had a short conversation with the researcher. All subjects returned for a second treatment session 2-3 days after the first visit. Subjects returned for the third and final treatment 2-3 days after the second treatment session. Immediately after the third treatment, the posttest battery was administered.

#### 2.3.3. Blinding

Subjects did not know whether they were in the treatment or placebo groups, and the researchers administering the laser treatments did not know whether they were using the treatment or placebo device. Appropriate laser safety goggles (Laservision, style F12, filter 000131.000) were worn by subjects and the researchers administering the laser treatment. The goggles had lenses rated OD 6+ @ 510–680 nm, which blocked all visible laser light. The goggles also hugged the face firmly, preventing laser light from entering from the side.

 To further avoid potential bias in the test results, the research team was divided into two groups. Two team members administered the laser therapy independently of two other team members that administered the battery of auditory tests. The members who administered the auditory tests also performed scheduling and group assignments. Groups were simply identified by colors; so, the testers did not know which treatment (laser, placebo, or control) any subject was receiving. The testers were not present during treatment sessions. The team members giving the laser therapy did not know which laser device was functioning and which was the placebo. A fifth member of the research team, not otherwise involved in the study, assigned the functioning and placebo laser devices to two of the color groups and was responsible for checking the devices weekly to ensure that the functioning laser device was working.

### 2.4. Laser Treatment Protocol

**Figure 1:**
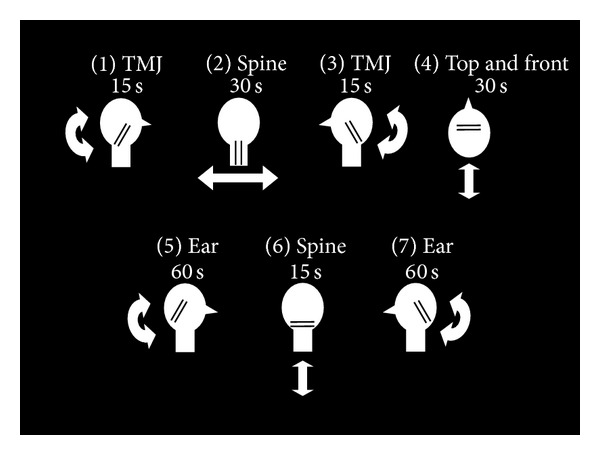
Visual depiction of the laser treatment. Each step is described in detail in the text. The white circle represents the subject's head. The double black lines represent the laser beams on the subject's head. The white arrows show the directional movement of the laser beams (horizontal, vertical, or rotational).

 The LLLT treatment protocol was based on a pilot study conducted by HearingMed (unpublished) showing improvement of word recognition scores following LLLT. Subjects in the treatment group had the low-level laser applied for approximately 4 minutes to the area around both pinnae, the back of the neck, and the top of the head. Subjects in the placebo group received the same protocol, except that the disabled laser device was used. Subjects in the control group simply sat in a comfortable chair and conversed with the research team member for a few minutes, and no treatment of any kind was administered. The laser was applied as described in the following steps and as shown in Figure 1.

**Step 1**

The laser was centered on the right temporomandibular joint, just anterior to the external auditory meatus of the ear, at a distance of approximately 2 inches from the surface of the skin. The hand-held probe was rotated from vertical to horizontal and back continuously for 15 seconds.

**Step 2**

The laser was positioned on midline of cervical spine with the beams running vertically from external occipital protuberance to the seventh cervical vertebrae. The hand-held probe was held at a distance of approximately 3 inches from the surface of the skin and continuously swept horizontally back and forth for 30 seconds.

**Step 3**

The left temporomandibular joint was stimulated, as described in Step 1.

**Step 4**

The laser was positioned on top of the head with the beams running across the head from ear to ear. The probe was held at a distance of approximately 2 inches from the surface of the head and continuously swept back and forth from the forehead to the occipital protuberance for 30 seconds.

**Step 5**

The laser was centered on the right external auditory meatus, with the probe held at a distance of approximately 2 inches from the surface of the pinna. The probe was rotated from vertical to horizontal and back continuously for 60 seconds.

**Step 6**

The laser was positioned over the cervical spine with the beams running horizontally. The probe was held at a distance of approximately 2 inches from the surface of the skin and continuously swept up and down from the occipital protuberance to the top of the shoulders for 15 seconds.

**Step 7**

The left external auditory meatus was stimulated, as described in Step 5.

### 2.5. Auditory Test Battery

The auditory test battery consisted of three assessments: pure-tone audiometry, speech understanding, and transient-evoked otoacoustic emissions (TEOAEs). These tests were chosen to examine different aspects of hearing; pure-tone audiometry assessed auditory sensitivity in quiet, speech testing assessed speech processing in noise, and otoacoustic emissions assessed the physiological state of the cochlea.

#### 2.5.1. Pure-Tone Audiometry

**Figure 2:**
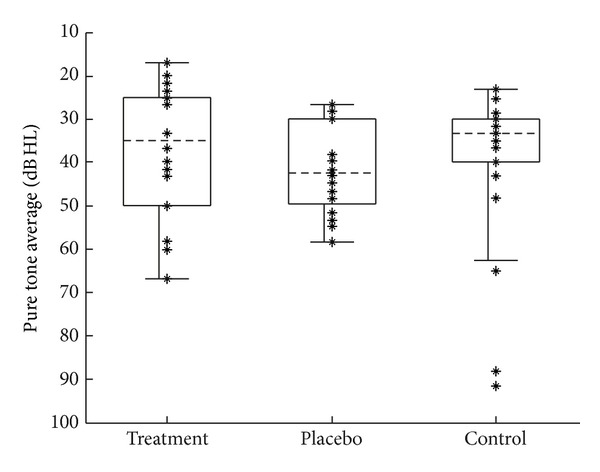
Distribution of pretest audiometric PTA for each group. The *y*-axis has been flipped to resemble an audiogram. Dotted horizontal lines indicate the median. Boxes demark the 25th and 75th percentiles. Asterisks show actual data points. (Data with identical values show as a single asterisk). Data from both ears of each subject are included, so that *N* = 18 (9 subjects), 20 (10 subjects), and 22 (11 subjects) for the treatment, placebo, and control groups, respectively.

**Figure 3:**
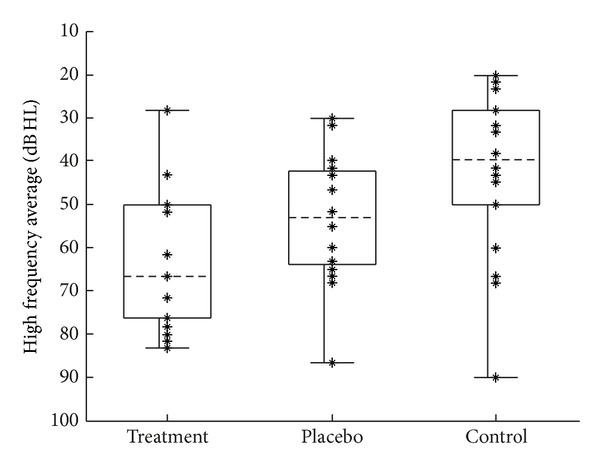
Distribution of pretest audiometric HFA for each group. Group means: 45.8, 44.8, 38.6 dB HL for treatment, placebo, and control groups, respectively. Figure format is the same as described in Figure 2.

Pure-tone thresholds were measured in 5 dB steps at six audiometric frequencies (0.25, 0.5, 1, 2, 4, and 8 kHz). Audiometry was conducted using custom software written in MATLAB (MathWorks) that implemented a method of adjustment psychophysical paradigm [16], with stimuli presented via ER-2 insert earphones (Etymotic Research). Thresholds for each subject were averaged to yield two measurements. The pure-tone average (PTA) was the average of the thresholds at 0.5, 1, and 2 kHz (Figure 2). The high-frequency average (HFA) was the average of the thresholds at 2, 4, and 8 kHz (Figure 3). 

#### 2.5.2. Speech Understanding

**Figure 4:**
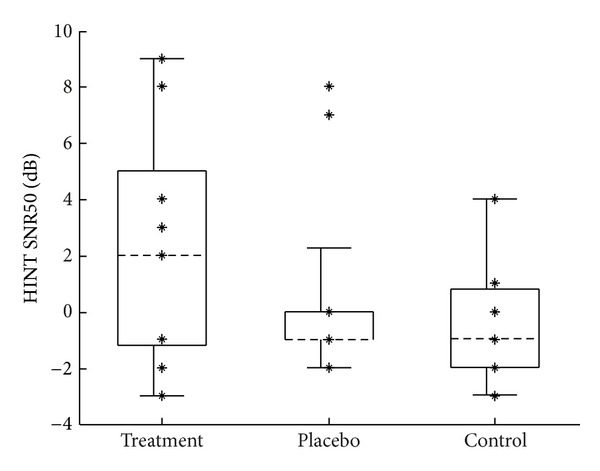
Distribution of pretest HINT SNR50 scores for each group. Group means: 2.1, 0.7, and −0.5 dB for treatment, placebo, and control groups, respectively. These values were used to set the signal-to-noise ratio for both the CST pre- and posttests. Figure format is the same as described in Figure 2.

The Hearing in Noise Test (HINT) [17] was used to determine the signal-to-noise ratios (SNRs) for the subsequent speech testing. This test is a prerecorded, adaptive measure of sentence speech reception thresholds in noise. Subjects are asked to repeat 20 sentences presented in a background of speech-shaped noise. For this experiment, the noise was fixed at a level of 65 dBA, and the speech level was adjusted adaptively based on the listener's responses. The speech presentation level across sentences 5 through 20 was averaged to obtain the level at which the listener achieved a 50% correct performance (SNR50). The SNR50 score obtained during pretesting (Figure 4) was used to set the SNRs for the Connected Speech Test (CST) for each subject, as described below.

 The CST [18, 19] provides objective quantification of the intelligibility of connected speech. The stimuli are a collection of passages about common topics. Each passage contains recordings of 9 or 10 sentences spoken by a female talker of average intelligibility. Subjects are asked to listen and repeat the sentences. Scoring is based on the number of key words repeated by the listener out of 25 key words per passage. The passages are embedded in multitalker babble. The SNR used in the present study was the SNR50 obtained from the pretested HINT, plus 4 dB. Scores were averaged across several test passages to produce a measure of intelligibility.

#### 2.5.3. Otoacoustic Emissions

Transient-evoked otoacoustic emissions (TEOAEs) are physiologic measures of the cochlea's response to a click-like acoustic stimulus [20]. TEOAEs were measured using an ER-10C probe microphone system (Etymotic Research). Transient stimuli containing energy from 1 to 8 kHz were used. A double-evoked paradigm [21] was used to cancel the stimulus and extract TEOAEs. Probe clicks were presented at a level of 87 dB peak-equivalent SPL, and the higher-level suppressor was presented 12 dB higher than the probe. Approximately 2000 recordings were made from each ear, and the recordings were averaged after artifact rejection. 

**Figure 5:**
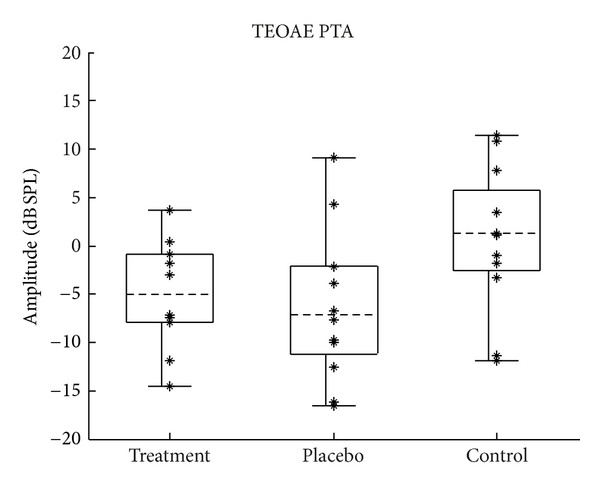
Distribution of pretest TEOAE PTA amplitudes (1-2 kHz). *N* = 10 (5 subjects), 12 (6 subjects), and 12 (6 subjects) for the treatment, placebo, and control groups, respectively. Figure format is the same as described in Figure 2.

**Figure 6:**
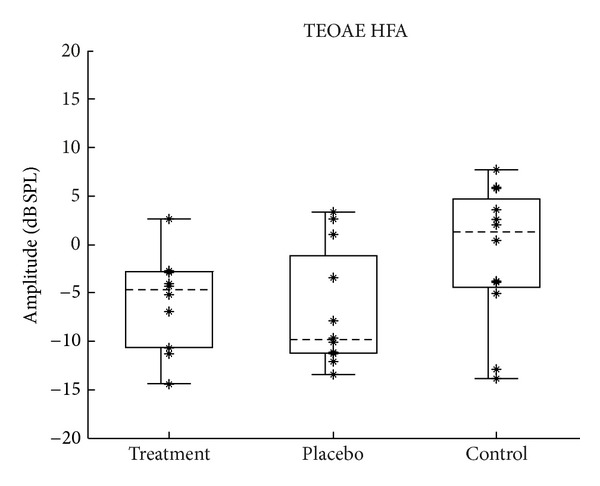
Distribution of pretest TEOAE HFA (2–8 kHz) amplitudes. Figure format is the same as described in Figure 2.

 TEOAEs were filtered into two bands: 1-2 kHz and 2–8 kHz. These frequency bands were chosen to correspond with the audiometric analyses and in this paper are referred to as the TEOAE PTA (1-2 kHz) and the TEOAE HFA (2–8 kHz). The distribution of pretest TEOAE amplitudes is shown for each frequency band in Figures 5 and 6. It was discovered during data analysis that some of the earlier subjects were tested using probe and suppressor levels that were much lower than intended. These subjects were therefore omitted from the data shown later and from subsequent analyses involving TEOAEs. These subjects' data were retained for the audiometry and speech test analyses.

## 3. Results

Data were obtained from both ears of each subject. Since no obvious differences were seen between left and right ears, data from both ears were combined in the following analyses. Strictly speaking, this likely violates the statistical assumption of independent sampling, since the test results from left and right ears of a single subject are likely to be highly correlated. None of the statistical tests used in the analyses are robust to the assumption of independent sampling, and the effect of including both ears is likely to be that of artificially increasing the sample size, making it more likely that a statistically significant result will be found. All statistical tests were conducted using a significance level of *α* = 0.05.

### 3.1. Pure-Tone Audiometry

**Figure 7:**
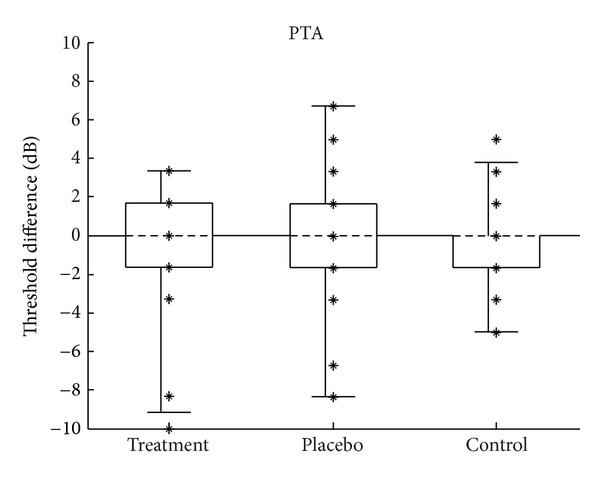
Change in the audiometric PTA. Change calculated as posttest minus pretest; negative values indicate improvement in thresholds. *N* = 18 (9 subjects), 20 (10 subjects), and 22 (11 subjects) for the treatment, placebo, and control groups, respectively. Figure format is the same as described in Figure 2.

**Figure 8:**
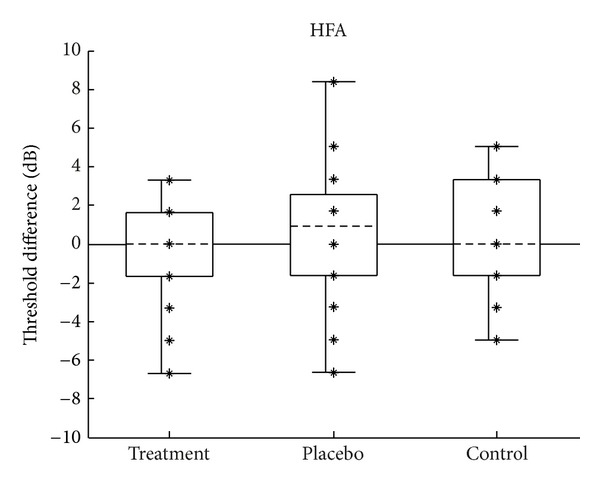
Change in the audiometric HFA. Change calculated as posttest minus pretest; negative values indicate improvement in thresholds. Figure format is the same as described in Figure 2.

Changes in the low-frequency pure-tone thresholds (PTA) were calculated by subtracting each subject's pretest PTA from their posttest PTA. Changes in the high-frequency pure-tone thresholds were computed in the same way using the HFA thresholds. Negative values indicated an improvement in thresholds after treatment, and positive values indicated a worsening. Figures 7 and 8 show the distribution of change in thresholds for PTA and HFA, respectively.

 Changes in PTA and HFA across the three groups were compared statistically. Analysis of variance was used to test the null hypothesis that the population means of the groups are all equal. Use of ANOVA requires four assumptions: (1) data are from groups with normally distributed populations; (2) data are from groups with equal population variances; (3) groups are independent; (4) data within groups are independent and randomly sampled. The test is robust to the first and second assumptions if the number of samples in each group is large and equal or nearly equal. The test is never robust to the third and fourth assumptions. The sample sizes in this data set (*N* = 18, 20, and 22) were probably large enough and close enough to the same size to meet the first two assumptions. Additionally, the sampled data did not suggest high skewness or kurtosis. The third assumption was assumed to have been met. As discussed at the beginning of Section 3, the fourth assumption was likely violated. 

 An analysis of variance showed no difference between group means for changes in PTA (*F*(2,57) = 0.09, *P* = 0.913) or for HFA (*F*(2, 57) = 1.33, *P* = 0.274). These results are shown in tabular form in Tables 2 and 3. 

### 3.2. Speech Understanding

**Figure 9:**
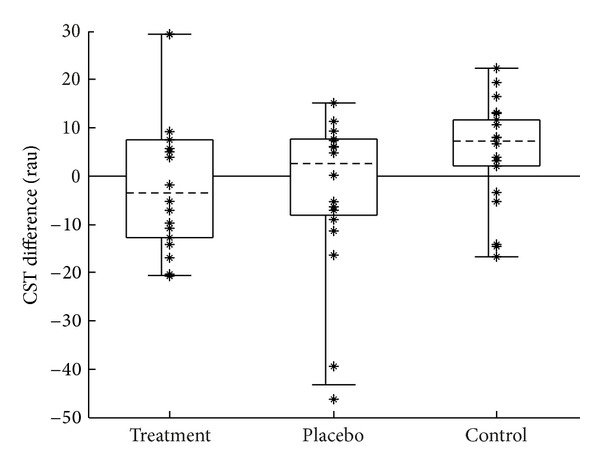
Change in CST scores expressed as rationalized arcsine units. Change calculated as posttest minus pretest; positive values indicate improvement in speech intelligibility. Figure format is the same as described in Figure 2.

Before computing changes in CST performance, scores were first transformed into rationalized arcsine units (rau) [19, 22]. Changes in rau scores were calculated by subtracting each subject's score obtained during the pretest from their score obtained during the posttest. Positive values indicate an improvement in speech understanding after treatment, and negative values indicate a decline.  Figure 9 shows the distribution of change in rau scores for each group.

 Changes in rau scores across the three groups were compared statistically. Analysis of variance was used to test the null hypothesis that the population means of the groups are all equal. The assumptions required by ANOVA were discussed previously. As they apply to the rau difference data, the sample sizes were probably large enough and close enough to the same size to meet the first two assumptions. However, the sampled data do suggest the possibility that the groups are differently skewed (sk = 0.471, −1.40, −0.55 for the treatment, placebo, and control groups, resp.). A Kruskal-Wallis test was therefore also performed to compare the medians of the groups. The Kruskal-Wallis test technically requires the assumption that the populations of the different groups are identical. The test is robust to all differences except differences in variability between groups. The test is reasonably robust to differences in variability if the sample sizes are equal. While the sample sizes in this data set were not exactly equal, they were close to the same. Additionally, the standard deviations, which are reasonable estimates of variability, were reasonably similar (SD = 13.06, 16.04, 10.55 for the treatment, placebo, and control groups, resp.).

An analysis of variance showed no difference between group means for changes in rau score (*F*(2,57) = 2.20, *P* = 0.120). The Kruskal-Wallis test showed no difference between group medians for changes in rau score (Kw(2) = 4.04, *P* = 0.133). These results are shown in tabular form in Tables 4 and 5. 

### 3.3. Transient-Evoked Otoacoustic Emissions

**Figure 10:**
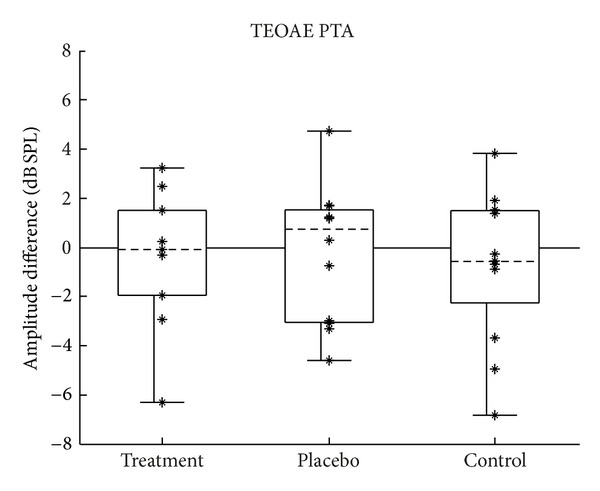
Change in TEOAE PTA amplitudes (1-2 kHz). Change calculated as posttest minus pretest; positive values indicate improvement. Figure format is the same as described in Figure 2.

**Figure 11:**
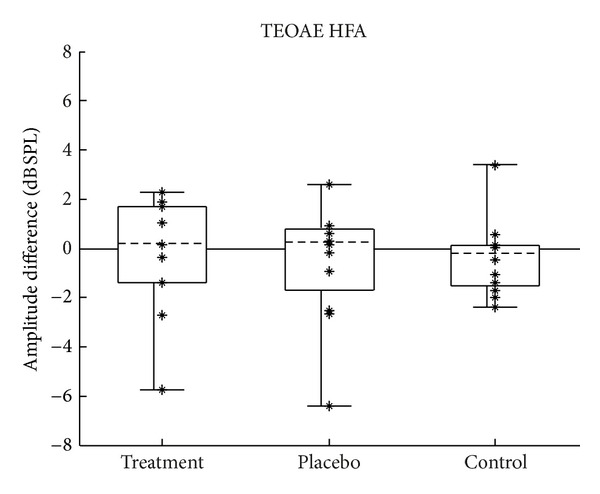
Change in TEOAE HFA (2–8 kHz) amplitudes. Change calculated as posttest minus pretest; positive values indicate improvement. Figure format is the same as described in Figure 2.

Changes in the lower-frequency TEOAE amplitudes were calculated by subtracting each subject's TEOAE PTA obtained during the pretest from their TEOAE PTA obtained during the posttest. Changes in the higher-frequency TEOAE amplitudes (TEOAE HFAs) were computed in the same way. Because TEOAEs are generated as a byproduct of outer hair cell function, significant positive values would theoretically be indicative of an improvement in outer hair cell function after treatment, and significant negative values would indicate a worsening. Figures 10 and 11 show the distribution of change in TEOAE amplitude for the lower and higher frequency bands, respectively.

 Changes in TEOAE PTA and TEOAE HFA across the three groups were compared statistically. Analysis of variance was used to test the null hypothesis that the population means of the groups are all equal. Regarding the assumptions required by ANOVA, the smaller sample sizes of the TEOAE data set were probably not large enough to make the test robust to the assumption of normality. The groups in the TEOAE PTA data set (Figure 7) appeared to be normally distributed and had equal variances (skew = −0.67, −0.06, −0.5; kurtosis = −0.47, −1.2, −0.8; SD = 2.8, 2.8, 3.0, for the treatment, placebo, and control groups, resp.). The third assumption was assumed to have been met. As discussed at the beginning of Section 3, the fourth assumption was likely violated. 

 There is no theoretical reason to expect the higher-frequency TEOAE data to be distributed differently from the lower-frequency data; however, the groups in the TEOAE HFA data set (Figure 8) were skewed, with the control group being skewed in the opposite direction to the other two groups (skew = −0.95, −0.94, 0.92, for the treatment, placebo, and control groups, resp.). Because of this, a Kruskal-Wallis test was also performed to test the null hypothesis that the population medians of the groups are all equal, in addition to computing a standard analysis of variance. 

 Analysis of variance showed no difference between group means for changes in TEOAE PTA (*F*(2,31) = 0.133, *P* = 0.876) or for TEOAE HFA (*F*(2,31) = 0.20, *P* = 0.819). These results are shown in tabular form in Tables 6 and 7. In addition, the Kruskal-Wallis test showed no difference between group medians for changes in TEOAE HFA (Kw(2) = 4.04, *P* = 0.133). These results are shown in tabular form in Table 8. 

## 4. Discussion

### 4.1. Clinical Significance and Statistical Power

None of the three measures of hearing (audiometric thresholds, speech recognition test, or otoacoustic emissions) showed a statistically significant difference between the treatment, placebo, or control groups. Although a statistically significant difference between the groups might be detected with a larger sample size, such statistical significance may not necessarily be clinically meaningful. As discussed later, no individuals showed any clinically significant changes on any of the auditory tests. 

#### 4.1.1. Pure-Tone Audiometry

From a clinical standpoint, a pure-tone threshold change of 10 dB or greater is generally considered significant [23]. Changes of a smaller magnitude are considered to be within normal clinical variability and are not suggestive of any significant alteration in hearing ability. As can be seen in Figures 6 and 7, changes in PTA and HFA were less than 10 dB for all subjects in all groups, and the changes were evenly split in the positive and negative directions. It is worth noting that a similar pattern was seen in individual frequencies: of the 300 audiometric measurements made (5 audiometric frequencies (0.5 to 8 kHz), left and right ears, 30 subjects), only one measurement showed a change greater than 10 dB. A single subject in the treatment group showed an improvement of 25 dB at 0.5 kHz in one ear. The other ear showed a 5 dB decrease. No improvement was seen in this subject's speech scores or otoacoustic emissions. The most plausible explanation is that the large threshold improvement was artifactual, in that the audiometric testing was always under the control of the subject. To summarize the audiometric test results, in addition to group means being the same, no individual subjects showed a clinically significant change in thresholds.

#### 4.1.2. Speech Understanding

When considering clinically significant changes in CST scores, it is necessary to know the critical difference of the scores, expressed in rau. Cox et al. [19] suggested that critical differences for this test should be derived from the measured within-subject standard deviation. They reported a 95% critical difference for hearing-impaired subjects of about 15.5 rau. Humes et al. [24] pointed out that the subjects in the Cox et al. study had a considerable amount of practice before the variability was measured. For the Humes et al. study, in which subjects received no practice with the CST, a 95% critical difference of 32.2 rau was determined. The present research is similar to the Humes et al. study, in that subjects received no practice prior to the data collection. Further, the standard deviation of the rau differences in control group of the present study was 10.55 rau, which is similar to the standard deviation of 11.5 rau found in the Humes et al. study. Examination of the distribution of rau scores for the control group (Figure 9) also supported using a 95% critical difference of somewhere between 25 and 30 rau.

 From Figure 9, it can be seen that there were three data points that exceeded a critical difference value of 25. Two of these were in the negative direction (suggesting a decrease in speech intelligibility), and one was in the positive direction. Since the critical value represents a 95% confidence interval, it can be expected that in any given sample, approximately 5% of the scores will exceed the critical value simply by chance. In the current sample, about 3 scores would be expected to exceed the critical value (0.05 ∗ 60 = 3). Further, these large changes occurred in only one ear of three different subjects, while changes in their other ears did not approach significance (−5.48, −11.56, and −5.49 rau). Finally, these large rau changes did not cooccur with significant changes in audiometric thresholds or TEOAEs. To summarize the CST results, in addition to group means being the same, no subgroup of individual subjects showed a clinically significant change in speech understanding.

#### 4.1.3. Otoacoustic Emissions

When considering clinically significant change in TEOAEs, an amplitude change of 6 dB or greater might be considered significant given the test-retest reliability in normal populations [25]. As can be seen in Figures 10 and 11, none of the measurements made a change of at least 6 dB in the positive direction, which would indicate significant improvement in TEOAEs. As with the other tests, in addition to group means being the same, no subgroup of individual subjects showed a clinically significant change in TEOAE amplitude.

### 4.2. Comparison with Previous Studies

As described in Section 1, conflicting results on the effect of LLLT on hearing have been reported. The current results are further evidence that LLLT does not have an effect on hearing status. In the current study, care was taken to blind the subjects, the researchers administering the treatment, and the researchers administering auditory testing. It appears that some previous studies were less careful about controlling for placebo effect and potential researcher bias. Future studies should also implement double-blinding, as well as control and placebo groups. Other factors may also explain the discrepancy in findings. Some previous studies may have achieved much greater laser penetration by using animal models [4] and isolated cochleae [5]. The current study, involving external irradiation of human subjects, likely involved less stimulation of structures associated with hearing. While a transmeatal approach to irradiation [7] would have achieved greater penetration, such an approach represents a less practical delivery method and is not commonly used by holistic practitioners of LLLT. Differences in laser wavelength and dosage may also contribute to variable results across studies. 

### 4.3. Limitations of Current Study

 As discussed previously, several statistical assumptions must hold true in order to report valid statistics (normality, equal variance, independence, and random sampling). Since the current study was intended to be a feasibility study, it was anticipated that by randomly sampling individuals with documented sensorineural hearing loss, some evidence of an intervention effect would be measureable, if any existed. Since no effect could be demonstrated across a number of outcomes for any individual subjects, the study was terminated. 

In this feasibility study, the timeline was fixed as per the pilot data from the manufacturer of the device. It is possible that the treatment number, treatment protocol, or even the duration of the entire data gathering was insufficient to show an intended effect. 

 The laser device was checked weekly, as per the manufacturer's guidelines. It is possible, though unlikely, that the laser diode or the total output power varied between subject applications, all of which took place within a 7–10-day period. 

## 5. Conclusions

 No statistically significant effect of LLLT on auditory function was found, as assessed by pure-tone audiometry, speech understanding, and TEOAEs. Additionally, no individual subjects showed any clinically significant change. It remains possible that other methods of LLLT could have an effect on hearing. Further research elucidating the anatomic and physiologic bases for therapeutic effects of LLLT on hearing are needed before further clinical testing is warranted.

## Figures and Tables

**Table 1:** Group characteristics.

Group	*N *	F/M	Age	PTA
Treatment	9	0.44	52.3 (14.6)	37.4 (15.9)
Placebo	10	0.30	58.9 (7.4)	41.4 (10.3)
Control	11	0.45	47.1 (16.7)	39.8 (18.6)

Total	30	0.40	52.8 (12.9)	39.6 (15.3)

N: number of subjects in group. F/M: female to male ratio. Age: mean age in years. PTA: mean audiometric pure tone average (0.5, 1, and 2 kHz) in dB HL. Numbers in parentheses are standard deviations.

**Table 2:** ANOVA for difference between audiometric PTA group means.

Source	SS	df	MS	*F*	*P*
Between	2.03	2	1.02	0.091	0.913
Within	632.95	57	11.10		

Total	634.99	59			

**Table 3:** ANOVA for difference between audiometric HFA group means.

Source	SS	df	MS	*F*	*P*
Between	24.78	2	12.39	1.326	0.274
Within	532.40	57	9.34		

Total	557.18	59			

**Table 4:** ANOVA for difference between CST group means, expressed in rationalized arcsine units.

Source	SS	df	MS	*F*	*P*
Between	782.61	2	391.30	2.204	0.120
Within	10121.90	57	177.57		

Total	10904.52	59			

**Table 5:** Kruskal-Wallis test for difference between CST group medians, expressed in rationalized arcsine units. This test was performed on scores transformed to ranks. The ranks assigned to tied scores were the average of the ranks those scores would have had if they were not tied. Adjusted scores (adj.) are also shown. The adjustment is based on the fact that the variance of the ranks is smaller when ties are present. Its use is justifiable in cases where ties can be assumed to be present in the population. Here, it is assumed that many of the ties are due to rounding. The adjustment is small unless there are large numbers of tied scores.

Source	SS	df	Kw	*P*	Kw (adj.)	*P* (adj.)
Between	123.08	2	4.036	0.133	4.063	0.131

**Table 6:** ANOVA for difference between TEOAE PTA group means.

Source	SS	df	MS	*F*	*P*
Between	2.18	2	1.09	0.133	0.876
Within	253.96	31	8.19		

Total	256.14	33			

**Table 7:** ANOVA for difference between TEOAE HFA group means.

Source	SS	df	MS	*F*	*P*
Between	1.92	2	0.96	0.200	0.819
Within	148.23	57	4.78		

Total	150.15	59			

**Table 8:** Kruskal-Wallis test for difference between CST group medians, expressed as rationalized arcsine units. The ranks were assigned as described in Table 5.

Source	SS	df	Kw	*P*	Kw (adj.)	*P* (adj.)
Between	160.32	2	1.617	0.446	1.617	0.446
